# Case Report: Implementation of stereoelectroencephalography in Kazakhstan: early experience in surgical planning for drug-resistant epilepsy

**DOI:** 10.3389/fnhum.2025.1666735

**Published:** 2025-09-29

**Authors:** Veronika Abzalova, Sholpan Kauynbekova, Gabit Makhambayev, Aleksandr Dmitriev, Berik Tuleubayev

**Affiliations:** ^1^Karaganda Medical University, Karaganda, Kazakhstan; ^2^Professor Makazhanov Multidisciplinary Hospital, Karaganda, Kazakhstan; ^3^Center for New Medical Technologies, Novosibirsk, Russia

**Keywords:** drug-resistant epilepsy, stereoelectroencephalography, multifocal, epileptogenic, neurosurgical evaluation

## Abstract

**Introduction:**

This clinical report describes the management of a 32-year-old patient with a long-standing history of drug-resistant epilepsy. It uniquely illustrates how stereoelectroencephalography (SEEG) played a significant role in the presurgical evaluation of a multifocal epileptic disorder which, despite a long history of no changes on MRI, was ultimately found to be associated with bilateral hippocampal sclerosis. This is one of the first documented cases of SEEG application in Kazakhstan, where the method was introduced in 2024.

**Clinical presentation and diagnostic findings:**

The patient suffered from debilitating seizures (4–6 times/week, often in series of 3-4/day) refractory to combined antiepileptic therapy. Scalp EEG revealed the first originating from the right frontotemporal leads with subsequent diffuse, predominantly right-sided, propagation. The second seizure, however, showed onset from the left temporal leads; notably, only left-onset seizures culminated in bilateral synchronization. Financial constraints precluding PET-CT and the diagnostic ambiguity of routine methods necessitated invasive SEEG.

**SEEG results and therapeutic strategy:**

SEEG monitoring unequivocally identified three independent epileptogenic foci: in the right hippocampus, left hippocampus, and left orbitofrontal region. Such multifocal pathology significantly reduces the likelihood of successful focal resection. Despite this inherent complexity, a crucial clinical outcome was achieved: the patient has remained completely seizure-free for 7 months following the ANT-DBS procedure.

**Conclusion:**

This report underscores the critical role of SEEG in the precise localization and characterization of complex, multifocal epileptogenic networks, often elusive to non-invasive modalities. It convincingly demonstrates that a comprehensive invasive approach can lead to successful seizure control even in cases previously considered inoperable. It also reflects the challenges and advancements in developing high-tech epileptological care in regions where advanced methods like SEEG have only recently been introduced.

## 1 Introduction

Brain cells function effectively through area- and function-specific electrical impulses that facilitate cognitive processes, perception, and adaptive responses to environmental and internal changes. Abnormal electrical activity in these cells leads to a disbalance of rhythms, provoking convulsive seizures and significantly increasing the risk of epilepsy. Globally, epilepsy affects approximately 50 million people, and the annual incidence varies significantly by region: in high-income countries, an estimated 49 new cases are diagnosed per 100,000 people each year, whereas in low- and middle-income countries, this figure can be as high as 139 per 100,000 (World Health Organization, 2024). Despite the existence of dozens of antiepileptic drugs, up to 30% of patients do not respond to pharmacotherapy, thus classifying them into the pharmacoresistant/ drug-resistant epilepsy category (Fattorusso et al., 2021).

Consequently, for this challenging patient cohort, surgical interventions become paramount in achieving meaningful seizure control.

This article presents a case of drug-resistant epilepsy where comprehensive preoperative assessment, including stereoelectroencephalography, was instrumental in determining the most appropriate surgical approach for this patient.

SEEG was introduced to clinical practice in Kazakhstan in 2024. Our practice aligns with internationally recognized indications for SEEG, which include the precise localization of the epileptogenic zone and the delineation of surgical boundaries for resection planning. Invasive evaluation is judiciously avoided in instances where clinical, electrographic, and imaging data consistently converge to support a cohesive hypothesis of seizure onset localization. Conversely, the presence of discordance among seizure semiology, EEG findings, and imaging studies renders invasive monitoring indispensable.

Currently, no published reports detail the implementation of SEEG procedures within Kazakhstan. This reflects a broader regional challenge; a recent study from Southeast Asia underscores the severe underutilization and limited availability of SEEG, with a ratio of merely one epilepsy center per 100 million inhabitants. Alarmingly, over 500 million individuals in the region reside in countries entirely devoid of SEEG access. Universal barriers to the expansion or establishment of SEEG services have been identified as human resource shortages and financial constraints, collectively contributing to a plateau in SEEG utilization across this geographical area (Fong et al., 2025).

SEEG provides a three-dimensional representation of the brain. This 3D understanding is crucial for localizing symptomatic, lesional and irritative zones, as well as defining the functional cortex. This methodology facilitates the acquisition of data regarding seizure initiation and propagation, alongside the interaction between the epileptogenic zone (EZ) and functional brain regions. Such insights are vital for precisely delineating resection limits and informing comprehensive surgical planning, including strategies for less invasive approaches or neuromodulation.

This concept, therefore, critically emphasizes the fundamental importance of investigating the spatial and temporal dynamics of electrical discharges, moving beyond a sole focus on the initial seizure onset.

## 2 Patient information

A 32-year-old patient presented with a long-standing history of illness since childhood. Over the years, he was followed by a neurologist and an epileptologist, underwent numerous diagnostic evaluations, and received prolonged pharmacotherapy. He was ultimately diagnosed with structural focal epilepsy, characterized by focal and bilateral tonic–clonic seizures (per ILAE 2017 classification), leading to a referral for neurosurgical evaluation.

**Figure 1:**
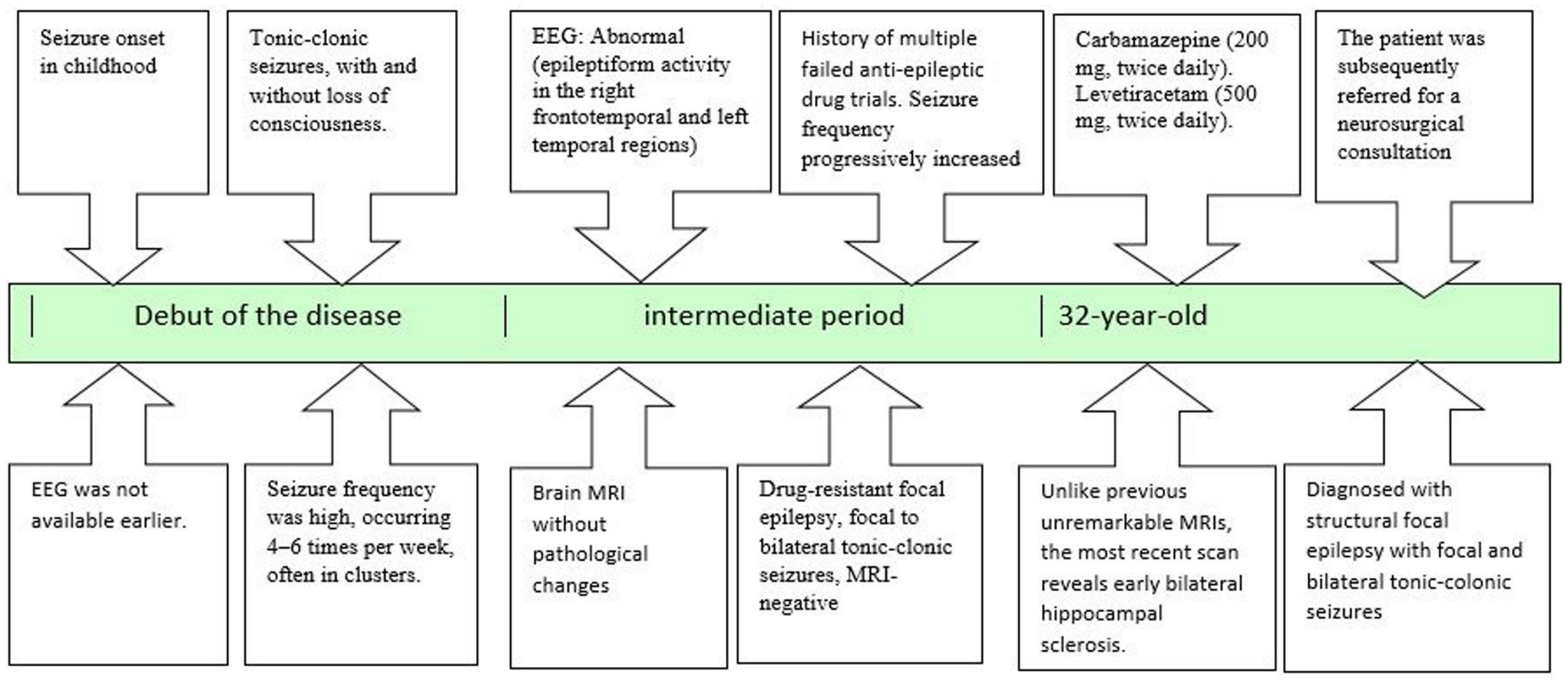
The patient’s journey, from initial diagnosis to neurosurgical referral.

A timeline detailing the stages from initial diagnosis to the neurosurgeon referral is presented in Figure 1.

## 3 Diagnostic evaluation

Scalp EEG analysis revealed distinct seizure onset patterns: the first originating from the right frontotemporal leads with subsequent diffuse, predominantly right-sided, propagation. The second seizure, however, showed onset from the left temporal leads. Notably, it was the left-onset event that terminated with bilateral synchronization of bioelectrical activity, indicative of a severe secondary generalization. The ictal scalp EEG records changes at the site of origin, and their effect on the clinical picture is consistent with the propagation of discharges.

The most recent magnetic resonance imaging (MRI) scan demonstrated bilateral hippocampal sclerosis. Crucially, these observed signs of sclerosis do not denote the initial onset of the disease but instead reflect pronounced neuronal degradation within the hippocampal formation.

Due to its high excitability and extensive connections, the hippocampus is susceptible to seizure activity. Recurrent epileptic discharges induce mossy fiber sprouting, which forms a new epileptogenic focus within the hippocampus and precedes hippocampal sclerosis. MRI visualizes only the end-stage of this process, without reflecting its duration and severity; moreover, the hippocampus can be involved either primarily or secondarily (Danzer, 2016).

The decision to perform SEEG was based on a combination of critical factors. A significant electro-clinical discordance was observed: scalp EEG recorded independent seizure onsets in both hemispheres, which contradicted atypical clinical features such as ipsilateral head version during seizures with both left- and right-sided EEG patterns. This discrepancy, coupled with the MRI finding of bilateral hippocampal sclerosis (BHS), presented a considerable diagnostic challenge.

Unlike typical unilateral temporal lobe epilepsy, BHS poses a more complex problem, as bilateral resection carries a high risk of severe anterograde amnesia. Considering this risk, along with the escalating seizure frequency, the failure of conservative therapy, and the unavailability of PET-CT, an invasive evaluation became necessary. The SEEG procedure plays a pivotal role in the precise differentiation between medial and lateral TLE, especially concerning the accurate delineation of the epileptogenic zone for potential resection. Therefore, an invasive diagnostic procedure was requisite for delineating the extent of the epileptogenic network and ascertaining the feasibility and scope of potential surgical intervention.

Temporal lobe epilepsy (TLE) is characterized by the involvement of a complex epileptogenic network that extends beyond the temporal lobe itself, often including the orbitofrontal cortex, insula, frontal and parietal cortices, temporoparieto-occipital junction, and other adjacent structures (Iida and Otsubo, 2017). In the United States, many centers favor ‘limbic’ coverage for SEEG implantation (Paredes-Aragon et al., 2022). Implantation typically targets temporal-insular-anterior perisylvian and/or temporal-insular-orbitofrontal regions, or posterior temporal-posterior insular, temporal-basal, parietal, and posterior cingulate regions. While various strategies exist, the practicality of such extensive or standardized implantation remains a subject of debate, with most neurosurgeons and epileptologists advocating for strictly individualized implantation.

### 3.1 General implantation strategy

**Figure 2:**
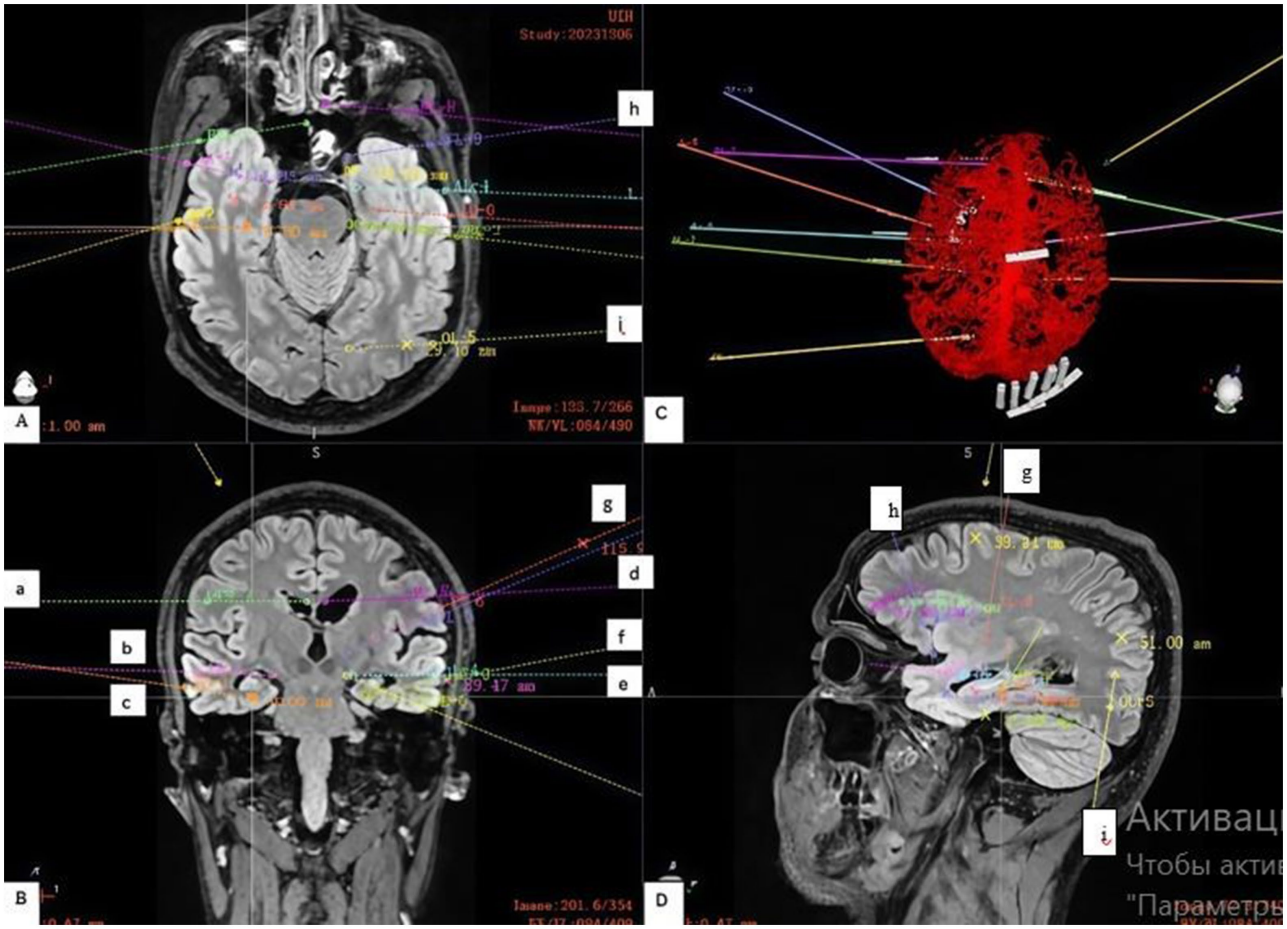
Stereoelectroencephalography (SEEG) Electrode Implantation Strategy. **(A)** Shows an axial view of the trajectories, targeting the: **(h)** left orbitofrontal cortex and **(i)** left occipital lobe. **(B)** Shows a coronal view with electrodes placed in key limbic structures: **(a)** right cingulate gyrus, **(b)** right amygdala, **(c)** right hippocampus; and **(d)** left cingulate gyrus, **(e)** left amygdala, **(f)** left hippocampus; and **(g)** left insular gyrus. **(C)** Demonstrates the planned electrode trajectories relative to the brain’s vascular structures, as confirmed by a contrast-enhanced computed tomography (CT) scan. **(D)** On the sagittal projection, the trajectory of the electrodes in the left hemisphere directed to the **(h)** orbitofrontal cortex, **(g)** insular gyrus; and **(i)** occipital lobe is more clearly visible.

Electrodes implanted bilaterally in the hippocampus (c, f), amygdala (b, e) and cingulate gyrus (a, d) formed the basis of the study (Figure 2). These three structures are key nodes of the mesial temporal (limbic) network. The presence of bilateral hippocampal sclerosis on MRI and temporal onset of seizures on EEG made it necessary to study these structures in both hemispheres. The aim was both to determine the independent onset of seizures in each hippocampus and to assess the possible role of the amygdala and cingulate gyrus as areas of early spread or independent foci.

Limited implantation in the right hemisphere was aimed at testing the hypothesis of its role, while denser coverage of the left hemisphere was necessary for detailed mapping of the more suspicious side.

Since the scalp EEG indicated a more complex and extensive network in the left hemisphere (only left-sided seizures led to generalization), additional key nodes were investigated (Figure 1).

Insular gyrus (g): Given the ability of insular seizures to mimic temporal or frontal seizures, as well as the patient’s complex semiology, examination of this deep structure was necessary to rule it out as the primary source of pathological activity.

Basal lobe/orbitofrontal cortex (h): Frontotemporal leads on the scalp EEG showed pathological activity. The orbitofrontal cortex has very close anatomical and functional connections with the mesial temporal structures (especially the amygdala). Seizures can spread very quickly between these areas.

Occipital lobe (i): The electrode was placed to determine the posterior boundary of the epileptogenic network. Given that hippocampal seizures often spread posteriorly along the temporo-parieto-occipital junction, understanding the posterior boundary of pathological activity is critical for planning a safe and effective operation (resection or neuromodulation).

The electrodes were implanted via a frameless robotic navigation technique utilizing the Remebot system. Pre-operative planning was meticulously executed, integrating high-resolution computed tomography (CT) and magnetic resonance imaging (MRI) data (Figure 2). Crucially, contrast-enhanced CT scans were incorporated into the planning phase to facilitate precise trajectory adjustments and mitigate the risk of vascular injury, which allowed us to successfully avoid damage.

Intracranial hemorrhage is a known complication during electrode placement. In an analysis of 201 SEEG implantations, evidence of hemorrhage was found in 23 cases (11%), or 0.9% of implanted electrodes. The full effect of these hemorrhages on epileptic activity has not yet been thoroughly evaluated, though contacts near intracranial hemorrhage often exhibit focal slowing with interictal epileptiform discharges or focal electrographic seizures (Agashe et al., 2023).

### 3.2 SEEG findings

**Figure 3:**
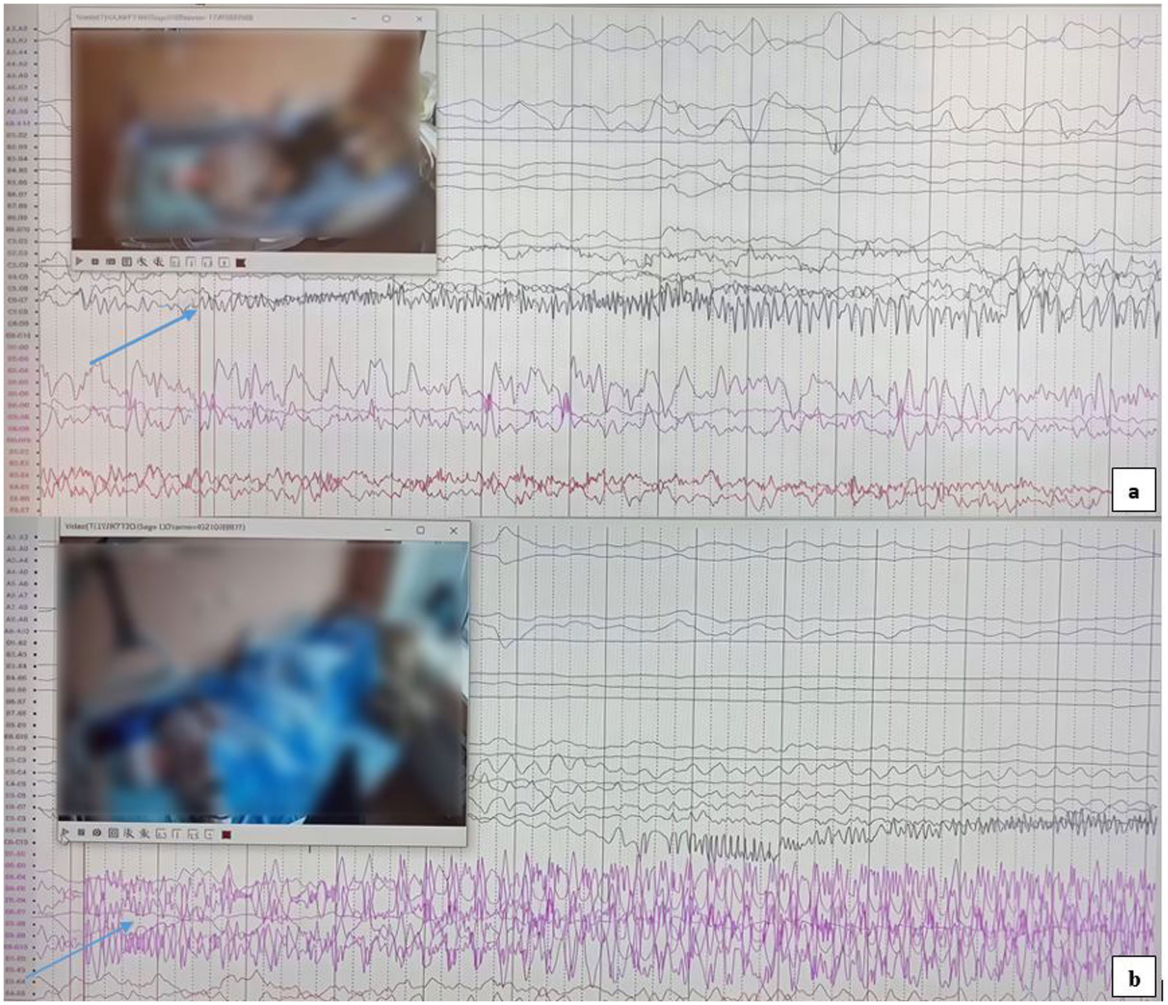
**(a)** Epileptic seizure activity originating from the right hippocampus, as indicated by the arrow. **(b)** The SEEG recording shows epileptic seizure activity originating from the left hippocampus, with the onset clearly marked by the arrow.

The first seizure began in the right hippocampus (Figure 3a) and subsequently propagated to the left hippocampus. It then spread to the right amygdala, likely via the anterior commissure and fornix. Finally, the discharge spread unilaterally to the left hemisphere, followed by bilateral synchronization. This rapid involvement of temporal structures is hypothesized to occur through extensive interconnections, including the commissura supracallosa, anterior commissure, and fornix.

The second seizure, originating in the left hippocampus (Figure 3b), was rapidly followed by bilateral synchronization. The swift propagation to the contralateral hemisphere suggests a short and efficient pathway for epileptic activity spread, likely involving the corpus callosum and thalamocortical connections. This phenomenon was ultimately characterized as an independent focus.

The third seizure was recorded in the left orbitofrontal region, definitively indicating the presence of multifocal epilepsy. This finding, in addition to the bilateral temporal seizure onsets, significantly reduces the likelihood of successful focal resection.

Analysis of the background activity revealed bilateral theta accentuation within the temporal regions, accompanied by the presence of sporadic sharp waves and spikes in the mediotemporal and anterotemporal leads. Non-specific indicator of widespread temporal lobe dysfunction. Furthermore, paroxysmal activity was consistently noted in the orbitofrontal areas.

Clinical manifestations varied across the recorded ictal events. The first seizure, originating in the right hippocampus, was characterized by an initial behavioral arrest, leftward gaze deviation, and right-hand dystonia. This evolved into right-hand automatisms, progressive disorientation, aphasia, and worsening speech and consciousness impairment. The second seizure replicated this semiological pattern, originating congruently in the left hippocampus. In contrast, the third seizure presented with a distinct clinical profile, exhibiting an abrupt onset from the left orbitofrontal region. This was marked by sudden grimaces, facial twitching, hyperexcitability, and transient vocalizations. The observed chaotic and less stereotypical behavior aligns with an orbitofrontal seizure onset, with probable secondary recruitment of limbic structures.

SEEG revealed structural lesions localized to both the left and right hippocampi, as well as the left orbitofrontal cortex. Concurrently, areas of irritation were mapped to the bilateral mediotemporal, anterotemporal, and orbitofrontal regions.

In light of these findings, neuromodulation, particularly stimulation of the anterior nuclei of the thalamus (ANT-DBS), became the most substantiated therapeutic solution, aimed at modulating pathological activity within the broad epileptogenic network without damaging functionally significant brain areas.

## 4 Therapeutic intervention

**Figure 4:**
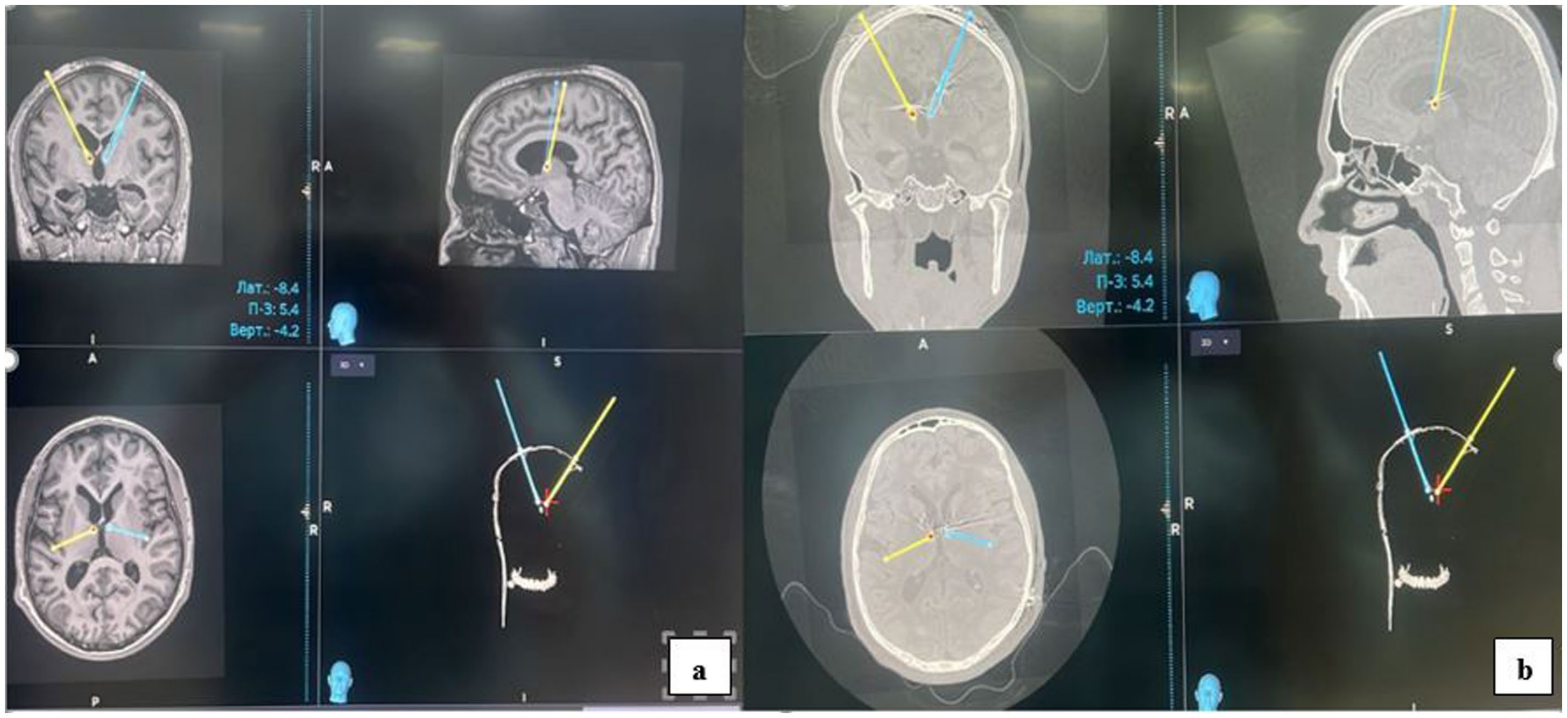
Postoperatively, electrode placement was confirmed by fusing postoperative CT scans **(b)** with the preoperative planning MRI **(a)**.

Electrode implantation for deep brain stimulation (DBS) of the anterior nucleus of the thalamus (ANT) was performed under general anesthesia. Preoperative planning utilized a Medtronic Stealth Station S8 neuronavigation system, integrating combined CT and MRI scans. The procedure itself was guided by the CRW® Stereotactic System. Postoperatively, electrode placement was confirmed by fusing postoperative CT scans (Figure 4b) with the preoperative planning MRI (Figure 4a).

## 5 Follow-up and outcomes

Over a 7-month postoperative follow-up, the patient has remained completely seizure-free. Stimulation parameters were programmed gradually, with initial settings at a conservative level (1.5 V, 90 Hz, 60 μs). At the most recent visit, stimulation was adjusted to 3.0 V, with other settings unchanged, which provided complete seizure control.

Given the early achievement of seizure freedom, the titration strategy was adjusted. Rather than proactively increasing voltage to the 5 V target recommended by the SANTE trial, we adhered to the principle of minimal effective stimulation to maintain the outcome and prevent potential long-term side effects (Fisher et al., 2010). The long-term risks of ANT-DBS, primarily depression and memory impairment, are well-documented by the SANTE trial (Salanova et al., 2015). Consequently, targeted clinical inquiry for these and other potential side effects was conducted at each visit.

During follow-up, the patient reported no side effects. For objective assessment, neuropsychological testing was performed before and 6 months after the operation, evaluating key cognitive domains (memory, attention, executive functions) and mood. The results revealed no clinically significant decline in scores compared to baseline.

## 6 Discussion

The presented clinical case is of dual significance. On one hand, it illustrates the diagnostic and therapeutic complexities associated with bilateral hippocampal epilepsy, requiring a thorough analysis within the context of global experience. On the other hand, it represents one of the first documented cases of SEEG application in Kazakhstan, allowing for an assessment of the novelty and potential of implementing this advanced technology in the region’s neurosurgical practice.

SEEG has profoundly influenced the modern approach to epilepsy treatment. Neurosurgeons and epileptologists are shifting away from the traditional search for an anatomical focus of pathological activity. Instead, epilepsy is now primarily conceptualized as a disorder of spatially distributed electrical discharges within a functionally connected neuronal network.

However, it should be noted that the currently available examination methods are unable to determine the epileptogenic area of the brain independently for several reasons. In certain cases of epilepsy, neuroimaging methods may not provide sufficient diagnostic information. In some instances, there is an absence of correlation between MRI changes and EEG activity. Of the 14 patients with both MRI- and PET-negative findings who pursued surgical treatment, 9 underwent resections after intracranial EEG evaluation. Notably, at the five-year follow-up, 77.8% of these patients demonstrated favorable outcomes (Pellinen et al., 2020).

Low concordance was observed between scalp and SEEG for seizure onset lateralization. A possible reason for such a discordant finding could be a delay between SEEG onset and its appearance on scalp EEG, which would allow the seizure to spread to the contralateral hemisphere (Gunnell et al., 2025). The discrepancy, which is considered rare in most studies, underscores the importance of bilateral SEEG electrode implantation for accurate seizure onset zone determination, particularly in cases where non-invasive methods yield ambiguous results. This is a crucial lesson for building an effective epilepsy surgery program in Kazakhstan, where non-invasive diagnostic resources may be limited or centralized.

The effectiveness of epilepsy surgery depends on complete removal of epileptogenic tissue while maintaining a functional and eloquent network postoperatively. Unsatisfactory seizure control may occur if the epileptogenic zone is only partially resected or if an additional focus not identified during preoperative evaluation (Kahane et al., 2006; Abzalova et al., 2024). Patients diagnosed with TLE typically represent a prime cohort for surgical resection. A recent meta-analysis revealed that SEEG confirmed unilateral TLE in 73% of patients initially suspected of bitemporal lobe epilepsy (BTLE) based on scalp EEG recordings. Crucially, 67% of these unilaterally confirmed patients subsequently experienced favorable outcomes (Engel Class I and II) following temporal lobectomy (Aghakhani et al., 2014; Di Vito et al., 2016). The profound impact of SEEG on surgical strategy is evident: a study comprising 21 TLE patients demonstrated that SEEG led to modifications in the initial surgical protocol for over 60% of cases (Dührsen et al., 2020). Moreover, data from various cohorts indicate that 20–45% of patients undergoing SEEG ultimately receive a substantially revised surgical plan (Punia, 2024).

In the present case, SEEG confirmed the presence of complex multifocal epilepsy with three independent foci and rapid propagation. Similar patterns have been described in previous SEEG studies of bilateral hippocampal epilepsy, where asynchronous or independent seizure onsets in each hippocampus complicate lateralization and contribute to lower seizure-free rates compared to unilateral mesial temporal lobe epilepsy (Steriade et al., 2019; Gonzalez-Martinez et al., 2013). Reported series indicate that, in true bilateral onset, Engel class I outcomes are achieved in only 25–45% of patients and the risk of postoperative memory decline, including severe anterograde amnesia, is high after bilateral resections (Dulay et al., 2006). Based on the prognostic data and the distributed network activity identified through SEEG, the decision was made to proceed with neuromodulation rather than resection. This risk–benefit assessment is particularly critical in the early stages of establishing a national SEEG program, where prioritizing patient safety and cognitive preservation can build trust and justify further investment in technology.

In summary, our experience highlights the transformative role of SEEG not only in defining the boundaries of a complex epileptogenic network, but also in determining the optimal surgical strategy. It is important to note that the SEEG methodology, developed in France as early as the mid-20th century (Talairach et al., 1974), has only become widespread in leading global centers over the last two decades, particularly outside of Europe (Jobst et al., 2010). Against this backdrop, the introduction of SEEG in Kazakhstan in 2024 is not merely a technological upgrade but a fundamental shift toward modern global standards in the treatment of drug-resistant epilepsy. This case demonstrates the feasibility of applying this technique within our regional context and serves as a foundation for the further development of a national epilepsy surgery program, including personnel training and the creation of local clinical protocols.

## 7 Patient perspective

I suffered from epileptic seizures for a really long time. My younger brother, who actually started his neurosurgery residency in 2024, he’s the one who brought me to this clinic. Back then, the doctors in the department were just starting to do this kind of procedure. By that point, I was honestly just so tired of the seizures that I agreed to the operation without a second thought. At first, I was pretty skeptical, of course. But I was so pleasantly surprised that this method genuinely works and that they actually perform such procedures here in Kazakhstan! I had visited so many clinics around our country before this, and they all told me that my epilepsy was too complex and that there was a high risk that removing part of the affected brain might not even help. Because of that, I just could not bring myself to take such a big step. But now… now I do not have seizures anymore. Though, I do not really want to talk about it too much, I do not want to jinx it.

## 8 Conclusion

Scalp electroencephalography remains a key routine method for formulating hypotheses regarding the epileptogenic zone. However, its diagnostic and localizing capabilities are inherently limited by multiple factors, including the size and depth of the ictal source, the degree of neuronal synchronization, and skull conductivity (Cosandier-Rimélé et al., 2007; Cosandier-Rimélé et al., 2011; Tao et al., 2007).

Positron Emission Tomography, by revealing the topography of glucose hypometabolism, demonstrates a high correlation with the epileptogenic network (Baumgartner et al., 2019; Kumar and Chugani, 2009). However, in the Republic of Kazakhstan, PET-CT is not currently covered by governmental healthcare programs, requiring out-of-pocket payment from the patient. Since PET-CT was unavailable to the patient due to financial constraints, invasive evaluation was required for further clarification of the epileptogenic zone.

Stereoelectroencephalography offers high spatial resolution, allowing for more accurate detection of restricted discharges and an improved signal-to-noise ratio due to a reduction in artifacts. Nevertheless, its utility is also constrained by the underlying hypothesis and the number of implanted electrodes.

In this context, the experience from this case allows for the formulation of several key conclusions for centers operating in similar conditions:

The pivotal role of multidisciplinary expertise. Success in complex cases is determined by the ability of the epileptology and neurosurgery team to jointly formulate a precise, hypothesis-driven implantation strategy.

Strict patient selection for maximum efficacy. In resource-limited settings, invasive evaluation should be applied to the most complex cases to guide the choice of the optimal strategy, thereby ensuring the best cost-to-outcome ratio.

Using successful cases as a tool for development. Currently, the stereo-electrode implantation procedure is fully covered by state funds, with no financial cost to the patient. However, the current volume of funding is a significant constraint; the number of implantations performed at our center does not even reach ten per year. In this context, especially considering the annual increase in the disease’s incidence, demonstrating a successful outcome in a complex patient serves as a powerful argument for the need to increase quotas and further investment. This case can serve as a precedent for developing national protocols and specialist training programs.

Ultimately, while diagnostic modalities are complementary, some may be mutually exclusive. Advancing technologies and understanding of epileptogenic networks introduce new therapeutic possibilities but also create new complexities. Enhanced precision in delineating epileptogenic zones, combined with a comprehensive multidisciplinary approach, is paramount for selecting the most appropriate and effective surgical intervention.

## Data Availability

The raw data supporting the conclusions of this article will be made available by the authors, without undue reservation.
